# 

**Published:** 1893-08-12

**Authors:** 


### CONTENTS.

| | PAGE |
|---|---|
| **The Work of the Hospitals Association** | 305 |
| **Around the Hospitals** | 306 |
| **Famous Poisoners in Fiction.** II.—A Daughter of Darkness | 307 |
| **Fighting the Microbe** | 308 |
| **Great Achievements under Suffering.** II.—The Blind Poet | 309 |
| **Contemporary Theory and Research** | 310 |
| **Annotations.**—The Dangers of "Safe" Hypnotics—The Sick-room—Do we Starve our Children? | 311 |
| **Notes and News** | 312 |
| **Scraps and Gleanings** | 320 |
| **The Book World of Medicine and Science** | vii |
| **Medical Vacancies, Appointments, &c.** | ix |
| **The Hospital Clinic—** | |
| CITY OF LONDON HOSPITAL FOR DISEASES OF THE CHEST, VICTORIA PARK.—Treatment of Pulmonary Phthisis. II. | 313 |
| BRISTOL ROYAL INFIRMARY.—The Treatment of Stone in the Bladder | 315 |
| NEW DRUGS AND PREPARATIONS.—Antitoxic Serum in Tetanus | 316 |
| **Editor's Letter-Box.**—Duodenal Indigestion | 316 |
| **The Institutional Workshop—** | |
| HOSPITAL CONSTRUCTION.—The Memorial Hospital, Mirfield | 317 |
| HOSPITAL MEETINGS.—The Hospitals Association | 318 |
| THE STORY OF THE INSANE FROM YEAR TO YEAR | 319 |
| CONSTRUCTION NOTES | 320 |

### EXTRA SUPPLEMENT.—THE NURSING MIRROR.

| | |
|---|---|
| **News from the Nursing World.**—The Royal National Pension Fund for Nurses—Cottage Nurses—Silent Sufferers—Nurses and Fire Drill—A Kind Suggestion—Advancement at Sunderland—Tailor and Nurse—An Important Suggestion—Every-day Dangers—An Unusual Comparison—Nurses at Chicago—English and American Training—Short Items | cxci |
| **Unprofessional Criticisms.** By A NURSE CRITIC | cxciii |
| **Use and Abuse of Uniform.** By A DISTRICT NURSE | cxciii |
| **Associations for the Supply of Cottage Nurses** | cxciv |
| **Male and Female Nurses in German Catholic Orders.** By Dr. KÖLLEN | cxcv |
| **Everybody's Opinion.**—The London Hospital.—Should Nurses be a Distinct Caste?—District Nurses! | cxcvi |
| **Midwives' Registration** | cxcvi |
| **Appointments** | cxcvii |
| **Novelties in Household Linen** | cxcvii |
| **Notes and Queries** | cxcvii |
| **For Reading to the Sick.**—The Best Bed-maker | cxcvii |
| **The Book World for Women and Nurses** | cxcviii |

